# A Rare Twist: Spleen Rupture in Plasmodium vivax Malaria

**DOI:** 10.7759/cureus.48546

**Published:** 2023-11-09

**Authors:** Sarah Alansari, Mouayad Abdulghani, Malik Zakaullah, Rambabu Vadlamudi

**Affiliations:** 1 Internal Medicine, Rashid Hospital, Dubai, ARE; 2 Emergency Department, Rashid Hospital, Dubai, ARE

**Keywords:** splenic infarcts, splenectomy, complicated malaria, spontaneous splenic rupture, plasmodium vivax malaria

## Abstract

Plasmodium vivax malaria is known for its recurring febrile episodes, typically considered less severe than its counterparts. This case study presents a distinctive case of a 22-year-old male from a malaria-endemic region, experiencing spleen rupture and infarction as rare complications of vivax malaria. We explore the uncommon aspects of this case, emphasizing the importance of early diagnosis and tailored management.

## Introduction

Despite substantial progress in malaria control, the disease remains a significant public health challenge, particularly in tropical regions. Plasmodium vivax, one of the causative agents of malaria, is characterized by its recurring nature. While often perceived as less severe than Plasmodium falciparum malaria, vivax malaria can manifest severe complications, such as spleen rupture and infarction. This case study delves into the atypical presentation, diagnosis, and management of these infrequent complications in a vivax malaria patient [1].

## Case presentation

A 22-year-old, previously healthy male from Pakistan presented to our emergency department with a chief complaint of sudden left-sided abdominal pain, which had been persisting for one day. He described the pain as localized to the left hypochondrium, non-radiating, and associated with intermittent fever and multiple episodes of non-bloody bilious vomiting. He mentioned that his most recent trip was six months ago, back to his home country.

Upon examination, the patient appeared pale and tachycardic, with a pulse rate of 100 beats per minute. His respiratory rate was 18 breaths per minute, and his initial blood pressure measured 148/99 mmHg. Physical examination revealed a rigid abdomen with tenderness throughout, more pronounced in the left hypochondriac region. While awaiting the results of blood tests, the patient became dizzy, and his blood pressure dropped. Intravenous fluids were promptly administered, and a focused assessment with sonography identified free fluid in the right hepato-renal and left spleno-renal spaces, along with inferior vena cava collapse.

Laboratory investigations revealed a hemoglobin level of 8.5 mg/dL, with a platelet count of 36,000 per microliter of blood. Elevated inflammatory markers were noted, with a C-reactive protein level of 173.7 mg/dL and a procalcitonin level of 97.96 ng/ml. A subsequent blood smear confirmed the presence of malaria vivax, with a parasitemia of 0.1% (Table 1).

**Table 1:** Lab results

Normal range	patient results
Hemoglobin (13-17 g/dl )	8.5 g/dl
Platelet count (150-410 10^3/ul)	36 10^3/ul
C-reactive protein (<5 mg/l )	173.7 mg/l
Procalcitonin (<0.05 ng/ml)	97.96 ng/ml

**Figure 1:**
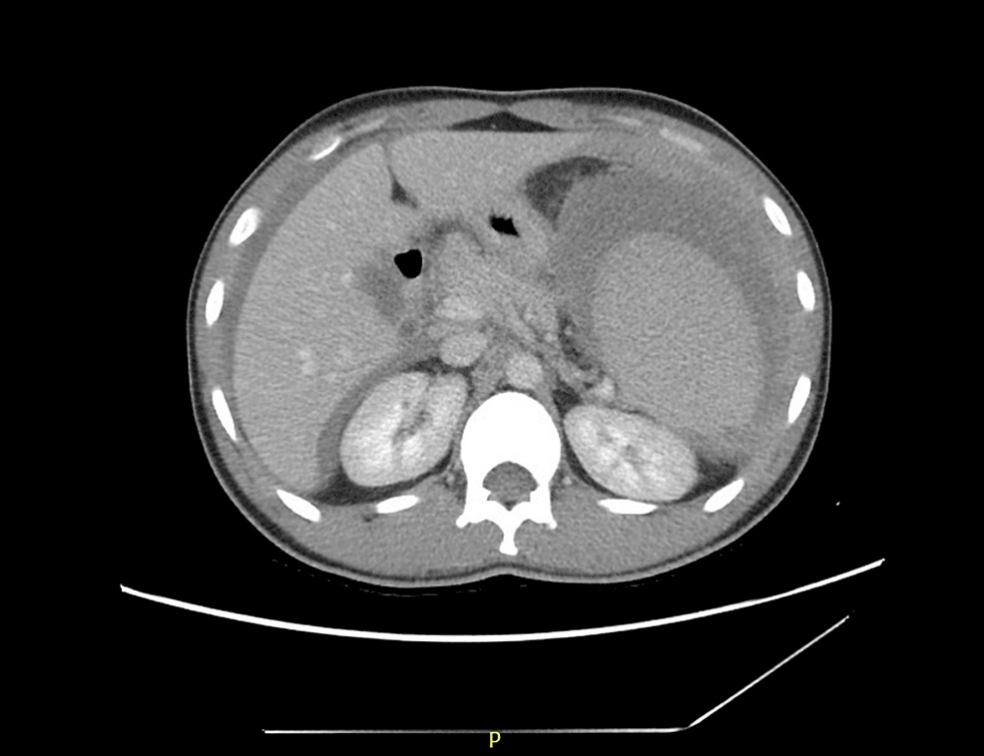
Computerized tomography of the abdomen Splenomegaly with free fluid in the abdomen with an average density of 30-40 HU suggestive of hemoperitoneum

**Figure 2:**
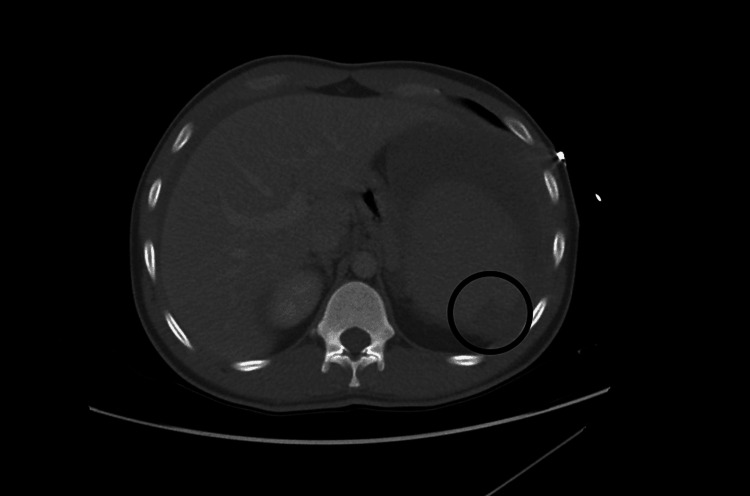
Computerized tomography of the abdomen Splenomegaly with a few peripheral wedge-shaped regions of hypo-enhancement suggestive of splenic infracts

After hemodynamic stabilization, the patient underwent computed tomography, which revealed splenomegaly with spontaneous rupture and multiple infarcts, leading to hemoperitoneum (Figures 1, 2). Due to his critical condition, he became a candidate for immediate surgical intervention. An emergency splenectomy was performed under general anesthesia, and histological analysis of the spleen specimen confirmed capsular disruption and intraparenchymal hemorrhage in keeping with splenic rupture.

Following surgery, the patient was transferred to the general ward, where he received close monitoring of vital signs, blood chemistries, and input-output measurements. He was initiated on antimalarial therapy with intravenous artesunate 2.4 mg/kg at 0, 12, and 24 hours. On the fifth day of hospitalization, he was discharged with a prescription for primaquine 30 mg daily for two weeks and received appropriate vaccinations, which included pneumococcal, Haemophilus influenzae type b, meningococcal, and seasonal influenza vaccinations.

## Discussion

Splenic rupture is an exceptionally rare yet critical complication that can occur during a primary acute attack of vivax malaria. In the existing body of literature, the majority of cases have been linked to infections caused by Plasmodium falciparum and Plasmodium vivax. Unfortunately, this complication remains underreported and often goes undiagnosed, with reported mortality rates estimated at approximately 38% among travelers and around 10% in indigenous populations residing in endemic regions [2].

Spontaneous splenic rupture represents a rare but highly lethal complication, with a mortality rate of 38%. The causes can be categorized into infectious and hematological factors, with infectious mononucleosis being the predominant infectious cause, followed by malaria infection.

The exact mechanisms behind splenic rupture during malaria infection remain unclear. Nonetheless, existing studies propose two primary hypotheses [3-4]: 

1. Sudden splenic congestion due to the engorgement of sinusoids by red blood cells, particularly in the presence of young trophozoites.

2. Microcirculation disorders caused by parasitized red blood cells, leading to localized disseminated intravascular coagulation.

The preferred therapy may differ among patients based on factors such as the severity of splenic rupture, the patient's hemodynamic condition, the accessibility of endovascular treatment, and the preferences of the attending physician. The primary goal of treatment should be the preservation of splenic tissue. For patients experiencing hemoperitoneum and ongoing instability, splenectomy continues to be the preferred treatment option [5].

We present a case study involving a 22-year-old male from an endemic area who exhibited signs and symptoms of splenic rupture concurrent with asymptomatic malaria infection. This observation may be attributed to repeated malaria infections over the course of his life, resulting in the gradual acquisition of immunity against the disease, subsequently leading to asymptomatic infections.

## Conclusions

This case underscores the importance of early diagnosis and immediate initiation of tailored antimalarial therapy in vivax malaria cases to avert rare complications, including spleen rupture and infarction. Healthcare providers in malaria-endemic regions should maintain vigilance in assessing and managing patients, recognizing the distinctive aspects of each case, and customizing treatment accordingly. Further research is needed to unravel the unique features of these rare complications and develop more effective prevention and management strategies.
